# A narrative synthesis scoping review of life course domains within health service utilisation frameworks

**DOI:** 10.12688/hrbopenres.12900.1

**Published:** 2019-03-29

**Authors:** Mary-Ann O'Donovan, Phillip McCallion, Mary McCarron, Louise Lynch, Hasheem Mannan, Elaine Byrne

**Affiliations:** 1 School of Nursing and Midwifery, Trinity College Dublin, Dublin, Ireland; 2 School of Nursing and Midwifery, University College Dublin, Dublin, Ireland; 3 Department of Epidemiology and Public Health Medicine, Royal College of Surgeons in Ireland, Dublin, Ireland

**Keywords:** health services utilisation, life course, framework, model

## Abstract

**Background: **Current thinking in health recognises the influence of early life experiences (health and otherwise) on later life outcomes. The life course approach has been embedded in the work of the World Health Organisation since the Ageing and Health programme was established in 1995. Yet there has been limited debate on the relevancy of a life course lens to understanding health service utilisation.

**Aim: **The aim of the review was twofold. Firstly, identify existing healthcare utilisation frameworks other than the dominant Andersen’s behavioural model currently in use. Secondly, to identify if current frameworks incorporate the advocated life course perspective in understanding health service utilisation.

**Methods: **A scoping review of PubMed, Cinahl Plus, Emerald, PsycINFO, Web of Knowledge and Scopus was conducted. Data extraction used a framework approach with meta-synthesis guided by the four domains of the life course proposed by Elder (1979): human agency, location, temporality and relationships, and interdependencies.

**Results: **A total of 551 papers were identified, with 70 unique frameworks (other than Andersen’s Behavioural Model) meeting the inclusion criteria and included in the review.

**Conclusion: **To date there has been limited explicit discussion of health service utilisation from a life course perspective. The current review highlights a range of frameworks that draw on aspects of the life course, but have been used with this perspective in mind. The life course approach highlights important gaps in understanding and assessing health service utilisation (HSU), such as utilisation over time. HSU is a complex phenomenon and applying a structured framework from a life course perspective would be of benefit to researchers, practitioners and policy makers.

## Introduction

The Global Health Strategy of ‘Health for All in the 21 ^st^ century’ strives for equality, quality and security of health access, while extending the number of healthy life years lived by all ( WHO, 1997). Within this strategy is a recognition that individual health experiences differ across and within populations, which ultimately results in divergent and unequal health outcomes that are linked to both medical and non-medical determinants of health ( WHO, 1997). The preceding Ageing and Health Programme 1995 laid the foundations for the prominence of a life course perspective in health, and as a central thesis of World Health Organization (WHO) activities, recognises the importance of understanding the impact of the life course on health and how health systems are developed ( WHO, 2000). This was further emphasized in the 2015 World Report on Ageing and Health ( WHO, 2015).

Aligned to this is the belief that health outcomes in later life are impacted by early life circumstances ( Braveman, 2014). This may be early-life health experiences, early life exposure to environmental, social, political and economic factors, or a combination of all of these early-life experiences in line with the life course view. The value of a life course approach in general is that it enables an understanding of the individual in relation to their environment ( Elder Jr & Rockwell, 1979), past and present.

Life and the individual as they exist at present is a product of when, where, how an individual has lived previously, the choices made throughout life and the people and relationships that formed part of that life. The individual is shaped by their historical, social, cultural and familial context, as well as the extent of personal control and choice experienced throughout his/her life. Elder (1994) proposes that a life course approach is one which incorporates four elements: location, agency or personal control, temporality and social relations and interdependencies ( Braveman, 2014; Elder, 1994; Harrison, 2003; Slota & Martin, 2003).

In this approach, the importance of early life events on later life outcomes and behaviours ( Braveman, 2014; Robison & Moen, 2000) is highlighted, as well as the relationships with family and dependencies and interdependencies with others over time. In addition, the extent of ‘agency’ or control a person has over their life is shaped by their environment (social, cultural, historical, locational and structural) and influences the direction and timing of life events and transitions.

It is all of these factors combined which form a life course perspective and this approach provides an opportunity to examine and address health, health inequities ( Braveman, 2014) and health service utilisation (HSU) across groups and across time.

The changing demographic profile globally, and within individual countries, impacts directly on the delivery of health services ( Goodyear-Smith & Janes, 2008), with the majority of developed countries facing the demands of an ageing population. Health service systems and the structure of service delivery needs to be informed by where and how the population is ageing to, as well as where and how the population has aged from. The supports and services that will be newly required or will continue to be used into the next stage of the life course will also inform these health service systems and delivery.

**Figure 1.**
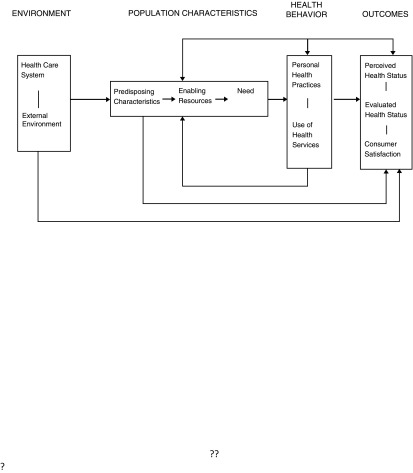
Andersen’s Behavioural Model of healthcare use. This figure has been reproduced with permission from ( Andersen, 1995).

The study of HSU is commonly explored using the Andersen Behavioural Model of Healthcare Use ( Andersen, 1995), depicted in Figure 1. The original Andersen model was proposed in 1968 with the focus on the use of formal personal health services, and the family as the unit of analysis. The purpose of the model was to help explain and predict use of health services. By the 1970s the unit of analysis had shifted from the family to the individual. Reference to the health system, to account for the role of health policy and its impact on health service use, was incorporated ( Andersen, 1995). There have been many adaptations of the core Andersen model across the years ( Goldsmith, 2007) and a number of reviews ( Babitsch *et al*., 2012; Guilcher *et al*., 2012; Phillips *et al*., 1998).

Although Andersen is the most frequently used model of HSU, it may not necessarily be the most appropriate framework for understanding HSU from a life course perspective. The Andersen model examines HSU at one point in time and there is no explicit recognition of the life course within this model. However, the generally accepted belief that health should be examined across the life course ( Elder Jr & Rockwell (1979)), would imply that to understand HSU a life course approach may be beneficial.

Thus, the purpose of this paper is that if we are to understand HSU more fully in light of transitions in living and choice, we need to identify:

1. What are the range of frameworks currently applied to the study of HSU?
2. Whether these frameworks are informed (or could be adapted to be informed) by a life course approach.

## Methods

For this scoping review, a methodical approach involving five steps of the systematic review process was followed to corroborate the paper objectives ( Briner & Denyer, 2012):

1.  Planning the review

2.  Identifying studies

3.  Evaluating study contribution

4.  Analysing the information

5.  Reporting accurate results

## Planning the review

### Approach

A narrative synthesis approach was used to identify and amalgamate the relevant framework information from appropriate studies for this paper. A narrative synthesis uses a textual rather than a statistical approach for analysing results and drawing conclusions. This words-based approach is more suitable to investigating the life course domains within HSU.

A scoping review is a descriptive review, which is a broader and less defined search than a systematic review. It is suitable for literature that is large, complex or heterogeneous. A scoping review can summarise the extent, variety and characteristics of findings on a specific topic, in this case the use of life course domains within HSU, and identify gaps in knowledge ( Tricco *et al*., 2018).

## Identifying studies

### Search strategy

The search spanned a 20-year time frame (1995–2015); 1995 was selected as the starting point for the review as this was the year the major revision of the Andersen model was published in the academic literature. Email alerts were set up in the databases to ensure the review was current. A summary of the review criteria is presented in Table 1.

**Table 1.** Summary criteria for review.

Aim	Identify frameworks used to understand/study health service utilisation framework from 1995–2015.
Participants	General adult population including vulnerable groups (minority, disease specific, disabled, intellectual disability, low income)
Interventions	Use of health services; accessibility of health services where accessibility refers to or leads to use of services
Outcome measure	Application of specific health services utilisation/health services accessibility model or framework
Study design	Any
Inclusion criteria	English language articles only; Full article availability only from 1995 to 2013; Specific named model or framework applied or developed to understand/examine health services utilisation.
Exclusion criteria	General health systems (strengthening) frameworks/models; Social determinants of health; Health inequalities/health disparity; Health delivery frameworks/models; Middle and low income countries; Health care workers

### Databases

PubMed, Cinahl Plus, Emerald, PsycINFO, Web of Knowledge, and Scopus were used for the search. The key search terms used were health services utilization OR health services utilisation OR health services accessibility AND (framework OR model); and related MESH terms in PubMed. The searches in Scopus and Web of Knowledge were restricted to journal title and included titles relevant to health services, health policy, health management, value in health, health planning.

An example of a search string that was used in PubMed for a custom year span of 1995–2015 is:

((((health service* utilization) OR health service* utilisation) OR health service* Access*) AND Framework) OR model

### Search procedure

**Figure 2.**
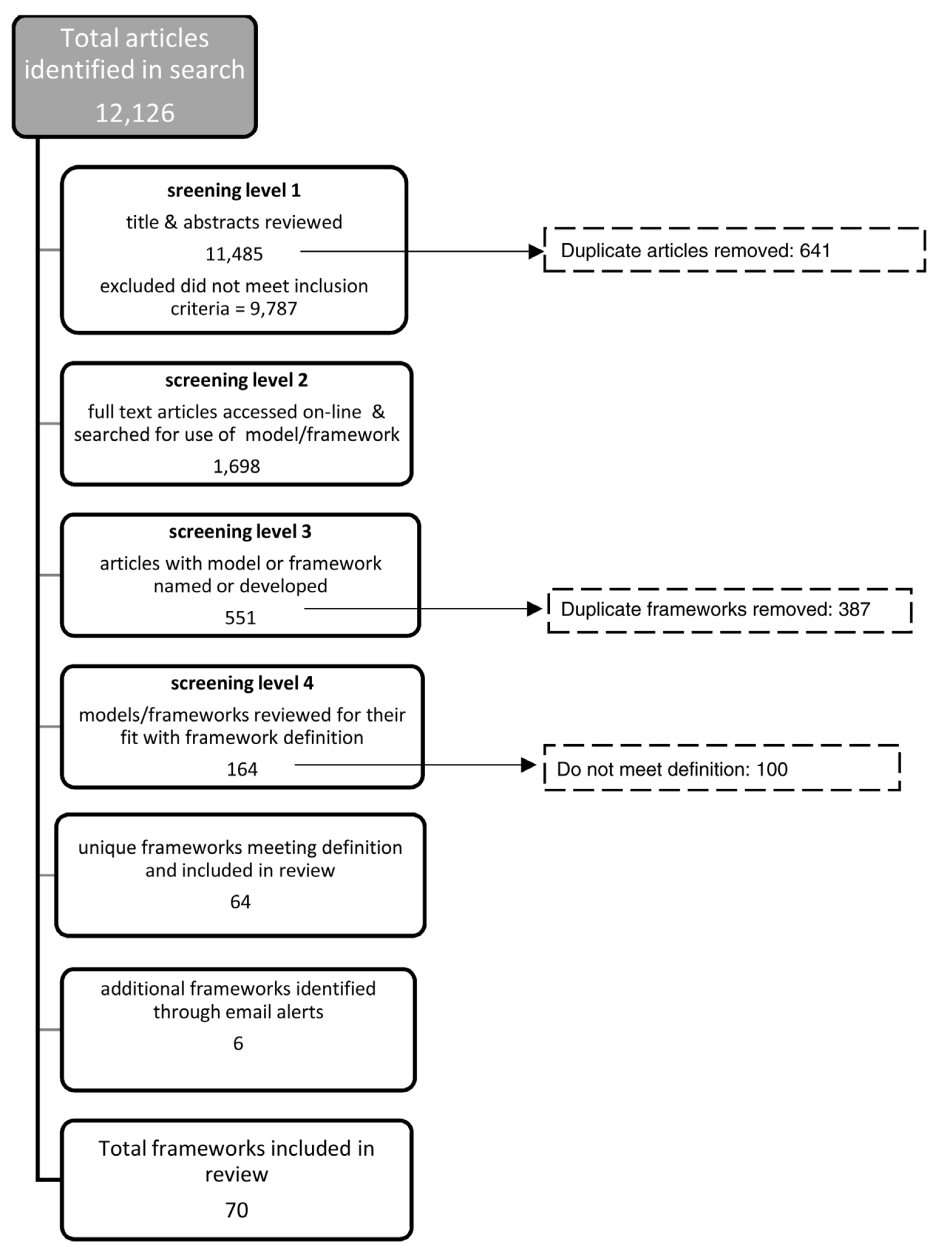
Flow chart for systematic review search of models or frameworks to study health service utilisation.

Application of the broad search string indicated above returned 12,126 articles. See Figure 2 for the flow diagram of results.

At stage one and two of the screening process, many articles were excluded based on the title, abstract or full text not meeting key inclusion criteria, leaving 1698 articles for further review.

The full text of the 1,698 articles were accessed and reviewed for the framework or model used in the study. Articles were excluded at this stage (stage 2) if statistical modelling alone as opposed to a conceptual or theoretical model for understanding health services utilisation was referenced, resulting in 551 articles remaining. If a framework was named or a new framework was proposed this was also recorded. As noted earlier only one article purposively selected with the occurrence of a framework was included. A total of 164 frameworks with an associated primary article remained after this third stage of screening.

To ensure that the identified frameworks were unique and separate frameworks, and were applied in an HSU context, each full text article (n=164), whether quantitative or qualitative, was read and confirmed for inclusion by MOD (and confirmed by EB). Further duplicates or adaptations of the Andersen model were excluded, resulting in 64 frameworks remaining. Six additional frameworks were subsequently identified through email alerts, and once reviewed against the same criteria, the final number of frameworks included in the review was 70. This list of frameworks was reviewed and consensus agreed for inclusion by the entire review team.

See Table 4 for a sample of the frameworks identified. The complete list of the 70 frameworks is available on Zenodo: https://doi.org/10.5281/zenodo.2590439 ( O’Donovan *et al*., 2019).

## Evaluating study contribution

### Frameworks

For the purposes of this review, the Behavioural model and any iteration will be referred to as the Andersen model/framework. The terms framework and model were used interchangeably and taken to be equal. Of note is that the context for use of the framework was to understand health services utilisation. Thus, the frameworks were identified as ‘used by researchers’, regardless of whether the original genesis or intent of the framework was to understand or examine this particular phenomenon. 

The frequency with which the identified models were used was not recorded, as the purpose of the review was to identify the range rather than prevalence/utilization of specific frameworks. Hence one paper using the identified framework was purposively selected for further analysis. These selected articles met the criteria for the systematic review most clearly and incorporated the most complete use of the framework. 

The unit of analysis was the individual’s use of services. The terms health services, utilisation and population are defined in Table 2. Inpatient, surgery and diagnostic testing were not included in this review.

**Table 2.** Definition of health services, utilisation data and population.

Health Services	Community based and primary care services, hospital-based therapy care (physiotherapy, Speech and language therapy, cancer care, chemotherapy; non anti-retroviral therapy care for people with HIV/Aids, dental services, emergency department, ambulance use, screening, preventative care, vaccinations and sexual health check screening)
Utilisation data	Use of the listed health services and accessing health services where it corresponds with use, visits, consultations, attendance, service consumption.
Population	People aged 18 years and over living in high income countries (based on World Bank listings)

### Screening

There were three levels of screening, led by the first author (MOD) with consultation meetings and review at each stage involving the entire review team (the remaining authors). The review team covered expertise across the areas of HSU (PMcC), health systems and qualitative research methods (EB), health and disability statistics (HM, PMcC) and intellectual disability and health (MMcC, PMcC). Each of these experts had previous experience in conducting systematic and scoping reviews. The review team systematically discussed the merit of each framework in relation to the aim of the review as well as the context of its application to understanding HSU. This process was on-going until a consensus was reached on the final list of frameworks for inclusion.

## Analysing the information

### Mapping

Once the frameworks were identified they were mapped against a matrix of HSU categories and variables. The Andersen model was the basis for ’a-priori’ categories and any variables not easily aligned with the Andersen model were categorised as ‘other specified’. The a-priori categories were the predisposing, enabling and need variables of the model. The rationale for this approach was to explore whether the Anderson model incorporated all the categories and variables of the other HSU models and if not, what these ‘missing’ variables were.

The Anderson model and the ‘other specified’ categories were then mapped to the life course domains of human agency, location, relationships and interdependencies, and temporality ( Elder Jr & Rockwell (1979)). Table 3 explains each of the life course domains. This mapping exercise explored whether the frameworks incorporated a life course approach and could therefore be used in exploring HSU from a life course perspective. See Table 5 for details of the mapping.

**Table 3.** Explanation of life course domains.

Life Course Domain	Meaning
Human Agency	Refers to an individual’s control, autonomy and choice in directing his/her own life path.
Location	Exposure to risks, whether the living environment is health-promoting and access to services can be determined by where a person lives
Relationships & interdependencies	Dependence (or not) on others for support to engage with a range of activities, functional, social, sexual, as well as support in asserting one’s own identity and place in the world,
Temporality	Historical effects on life/the timing of lives

**Table 4.** Sample of frameworks identified and purposive sample of articles.

No.	Framework/model identified	Description	Sample article from current review
1	Access Triangle	This framework specifically focuses on access to dental care for underserved populations.	Guay, 2004; 135(11):1599–605
2	Cooper model of access to care	Copper model explores barriers and mediators to health service use across personal, structural and financial aspects	Messer *et al.*, 2013
3	Frenk’s domains of Access	“Frenk reserves the term access to denote the ability of the population to seek and obtain care”	Levesque *et al.,* 2013
4	Gullifords model of healthcare access 2001	Gullifords model of access depicts relationship between factors and how they impact on access. This model was adapted by Alborz *et al*. for people with ID.	Alborz *et al.,* 2005;
5	Hispanic Farmworker Health Model ( Ward, 2007)	The HFH model incorporates 4 determinants of health and 2 outcomes specific to farmworker of Hispanic ethnicity. Highlights importance of living and working conditions and social and cultural factors	López-Cevallos *et al*., 2013;.
6	ICF framework	The authors used the ICF “was chosen because it allows examination of personal, environmental, and contextual factors, as well as impairments, activity limitations, and participation”.	Iversen *et al.,* 2011; WHO, 2001.
7	Modified model of access to dental care	This model is combination of elements of Penchansky & Thomas (1981) and Maxwell (1984) models to understand access to dental services by PwID.	Owens *et al.,* 2010;
8	Penchansky & Thomas 1981 Concept of Access: definition and relationship to consumer satisfaction	This model introduces the concept of ‘fit’ between the individual and the service. Five dimensions considered important – availability, accessibility, accommodation, affordability, and acceptability.	Wallace & Macentee, 2012.
9	Complementary and Alternative Medicine (CAM) use – a conceptual model	Draws on Parson’s sick role theory, Suchman’s stages of illness theory and Andersen’s Sociobehavioural Healthcare utilisation Model to examine use of complementary and alternative medicine.	Davis *et al.,* 2011
10	Non-urgent Emergency Department use – a conceptual model	Conceptual model of pathway to use emergency department	Uscher-Pines *et al.,* 2013;

Note: N=2200 approximately if included all 70 models

**Table 5.** Meta-synthesis of variables.

Andersen	Variables shared with other frameworks	Variables unique to other frameworks	Alignment to life course themes
***Environment***			
*Health care system*	Equity of services; Decreased disparity; Healthcare reaching; Health consequence; Accommodation; Accessibility; Quality of providers; Experience dealing with People with Intellectual disability (PwID); Willingness to serve; Liaison; Referral by medical practitioner; Reinforcing factors		Location Relationships & interdependencies
*External environment*	Local advertising ; Cues to action; Alleviating stigma	living conditions - physical, biological, social, cultural, material; traffic congestion/courtesy,	
***Population***			
*Predisposing*	Personal attachment style; Patience; Intrinsic motivation; Cognitive appraisal; Affective response; Self-confidence; Level of intellectual disability; ability factors at individual and family level; cognitive level; Cultural Beliefs and matching; Feminism; Readiness to change; Assumption of sick role; Equity in health; Disposition to act; Health competence; Health knowledge; Awareness and information factors; Perceived threat; Planning; Ethnic inequity;		Relationships and interdependencies
*Enabling*	Mother - age, race, marital status, education, employment status; if child is in or out of house; others - functional ability, burden; future worry); availability of carer/advocate; Relationships; Family separation; Advocacy; Cultural distance, socialization experience; Folk arena; Social inclusion; Social interconnectedness; Social capital – individual and community level; Sick person requires assistance to get better; Human rights; Person, behaviour, environment interaction; Resources (monetary, incentives);		Relationships and interdependencies
*Need*			
***Health behaviour***			
*Personal health* *practices*	Avoidance;	Life/lifestyle choices; Adherence;	Human agency
*Use of health* *services*	Multiple types of use options;	intention to use service; utilisation propensity; unmet need; delay or no treatment; ability to get care (perceive, seek, access);	Human agency Temporality
***Outcomes***			
		employment outcome;	
*Perceived health* *status*	Outcome expectancy/efficacy; Inaccurate symptom interpretation;		
*Evaluated health status*	Disease activity index (RADI); Multi-dimensional health assessment questionnaire (MDHAQ); Perceived susceptibility; Days hospitalised; Disability days; Efficacy of treatment; Health outcomes at individual and community level;	Care appropriateness,	
*Consumer* *satisfaction*			
		Life events; Life changes/structure; Permanent change or modification of life situation; Life disclosure; Time;	Temporality
		Perceived control; High or low control of services; Agency; Decisional control; Informed choice and decision-making	Human agency
		Choosing/appraising; Decision to seek help; Process component - danger control/fear control; Fear Cost/benefit analysis; Competing priorities	Human agency
		Message – acceptance/rejection;	Human agency

This process of meta-synthesis was conducted by two of the authors (MOD and EB) and reviewed by the entire review team. It involved a process of sense-making and developing a cohesive structure with which to present the findings of the extraction.

## Results

In total 29 of the 70 frameworks identified were specific HSU frameworks, with the remainder drawing on models from within psychology, sociology, disability studies and human rights, and applied to a HSU context. The frameworks are now discussed across the four life course domains. See Table 5.

*1. Human agency*

Human agency refers to the individual as an actor who plans and makes choices in their lives ( Elder, 1994). In the context of this review, agency was identified in terms of choice and control. Fifteen frameworks indicate the role of choice in HSU through the inclusion of variables of lifestyle choice ( Alborz *et al*., 2005), individual decisions and decision points, and the environment ( Stoller *et al*., 2011). This is described well in the work of Stoller *et al*. (2011), which is framed by Leventhal’s concept of the individual as an active problem solver in relation to their health, and Cockerham’s healthy lifestyle paradigm which includes agency and life chances (structure), and the interaction between both of these ( Leventhal *et al*., 2003; Stoller *et al*., 2011).

The individual as self-directing is also captured by McCormack & Mackintosh (2001) in the conceptualisation of pathways to health. The concept of decision-points in relation to use or selection of services ( Engelman *et al*., 2011; Shaw *et al*., 2013; Uscher-Pines *et al*., 2013), informed choice ( Essink-Bot *et al*., 2013), ability to seek care ( Levesque *et al*., 2013) and individual involvement in decisions ( Lee *et al*., 2009) were also identified.

Control features in a more limited way than choice in the reviewed frameworks with six instances of control specifically mentioned: decisional control ( Witucki & Wallace, 1998), low job control and how this is related to stress ( Azagba & Sharaf, 2011), health locus of control ( Davis *et al*., 2011) and perceived control ( Jo *et al*., 2008; Wong *et al*., 2006). Hubbell (2006) incorporates reference to danger control and fear control. Concepts of cost/benefit analysis were identified in the work of Fonseca-Becker *et al*. (2010) and Iversen *et al*. (2011) and imply a specific type of decision around competing priorities that an individual may make.

Choice and control were not identified in the Andersen model. The individual as an active participant in their lives, could be said to incorporate attitudes, beliefs, values and knowledge ( Davis *et al*., 2011; Hubbell, 2006; Jo *et al*., 2008; McCormack & Macintosh, 2001; Wong *et al*., 2006), internal processing ( Davis *et al*., 2011; Essink-Bot *et al*., 2013; Shaw *et al*., 2013), and disposition to act ( Essink-Bot *et al*., 2013; Fonseca-Becker *et al*., 2010; Leventhal *et al*., 2003; Levesque *et al*, 2013; Stoller *et al*., 2011; Wong *et al*., 2006), which inform the decisions made and actions taken. These could be captured under the predisposing element of the Andersen model and were identified in 14 frameworks.

*2. Location*

Six frameworks incorporated a specific place variable, which included urban/rural distinction Hubbell, 2006; Seccombe, 1995; Stoller *et al*., 2011), geographic location ( Levesque *et al*., 2013), living and housing conditions ( Koopmans & Lamers, 2007; López-Cevallos & Garside, 2013; Ståhlnacke *et al*., 2005), residential settings ( Essink-Bot *et al*., 2013) and distance to travel and neighbourhood factors ( Birkin *et al*., 2008; Graves, 2009; Meade *et al*., 2015; Mobley *et al*., 2006). Essink-Bot *et al*. (2013) specifically refer to living in a residential setting but this is the only occurrence of such a place variable in the frameworks.

Transportation ( Messer *et al*., 2013; Shaw *et al*., 2013) and service setting ( Alborz *et al*., 2005) are other location variables identified as influencing HSU. Toloo *et al.*. (2013) includes living status as a socio-demographic variable to consider in HSU. In this context, it refers to if the person lives alone or with someone else (partner, children, family etc.), thus highlighting the link between place and people ( Toloo *et al*., 2013) and the role of relationships in HSU.

Andersen does not explicitly mention location in terms of geographic, physical and social make-up of the space within which an individual lives. Travel to health service location is incorporated within Andersen as an enabling factor.

*3. Relationships and interdependencies*

The relationships and interdependency domain, or as otherwise referred to by Elder (1994) as linked lives was identified in 20 of the reviewed frameworks. The importance of social interconnectedness ( Levy *et al*., 2014), psychosocial ( Nauenberg *et al*., 2011) and affective supports ( Witucki & Wallace, 1998) to HSU were highlighted.

Communication and rapport with the healthcare provider was identified in the context of immigrants who were non-English speaking ( Jo *et al*., 2008). Broader issues of human rights ( Gibson & Mykitiuk, 2012), feminism ( Bergeron & Senn, 2003), stigma ( Yalon-Chamovitz, 2009), and legal status ( Hvinden, 2003) are included within this domain. This is because they impact on the status and roles an individual has within a given society and how others in society interact with the individual. Cultural beliefs ( Barrett *et al*., 2003) and folk values ( Thurston *et al*., 2014) also represent a type of interdependency that was recognised in some frameworks as impacting on HSU.

Relationships and interdependencies manifest in the Andersen model under the enabling factor of personal and family means to access services and availability of health personnel, as well as the social networks and interactions under the predisposing variable.

*4. Temporality*

Temporality within life course theory recognises the impact of when people are born, and societal and historical context within which the person is born, on their life course trajectory ( Elder, 1994). Implicit within the concept of temporality of the life course, is that of change over time and the differing experiences and timings of change and transitions. With this, the current review identified temporality as any time-related variable or variables linked to change occurring, that have time implicit in the concept.

Changes in the life course were identified in a small number of the reviewed frameworks (n=4). Stoller *et al*. (2011) include life changes/structure, while Petersen (1990), as referenced in Ståhlnacke *et al*. (2005), includes permanent change or modification of life situation in his conceptualisation of health-related behaviour.

Life events were identified in two frameworks, with Setia *et al*. (2011) referring to Berry’s (1997) life events and Kosteniuk & D’Arcy (2006) referring to early life as an important consideration for understanding utilisation of health services. Time as a factor was included in the conceptual model by Xiao *et al*. (2014). This was the only explicit occurrence of time in the frameworks.

The Andersen model presents a snapshot of health service utilisation and does not incorporate a change or time variable.

The PRIMSA-ScR checklist, PRIMSA flow diagram and Table S6 which contains all 70 frameworks identified in this review are available on Zenodo: https://doi.org/10.5281/zenodo.2590439 ( O’Donovan *et al*., 2019).

## Discussion

### Andersen and the life course

Andersen’s framework ( Andersen, 1973; Andersen 1995) provides a useful basis for understanding and exploring predictors of health services utilisation. This is illustrated by the frequent application and adaptation of the framework to different health service contexts. In addition to the application of the Andersen model, which is the dominant guiding framework in the study of HSU, this review indicates that HSU in some contexts, with some populations and specific service settings is not sufficiently addressed through the Andersen model. This is evidenced by the emergence of such a range of alternative frameworks.

When assessing HSU in the context of the life course theory, Andersen is limited in its incorporation of the domains of the life course. Most notably, through the absence of issues related to human agency and temporality, limited reference to location, and some engagement with relationships and interdependencies. Though the traditional HSU model does not include core domains, recent reviews of the model indicate that the application of the model in this way has begun. For example, the domains of choice, decision-making and control are not explicitly evident in the Andersen framework, but the health locus of control concept was noted by Babitsch *et al*. (2012) as used in one study as a predisposing factor. This shows the potential to adapt the model to take on aspects of a life course perspective.

The particular strength of the Anderson model is very useful for considering the individual’s interaction with the health system at one point in time for one condition/scenario. Temporality as envisaged within the life course is not addressed within the model, although ‘time interval’ does feature within phase two of the model. This time interval refers to time within the health system, such as waiting time. However, the alternative frameworks propose ways to capture the temporality of health and temporality of health care need and use over the life course.

Strong across both Anderson and non-Anderson models, is the recognition of the importance of systems of support around the individual to engage with and access the health system. Relationships and interdependencies are how this is relayed in the life course approach and it is apparent in both.

Andersen identifies family and community level variables as important enabling resources to HSU with social and emotional supports recorded by Babitsch *et al*. (2012). Guilcher *et al*. (2012) recommend the inclusion of quality of relationships/emotional support with informal and formal carers to future adaptations of Andersen. Andersen introduces the concept of ‘site’ of the health service in version two and ‘external environment’ in version three of his model, again related to the health system, and are location-related variables ( Andersen, 1995).

Applications of the Andersen model have used broad place variables at the level of geographic location and community characteristics such as distance travelled, urban/rural distinction and region. There is an absence of a more refined place variable such as type of living setting, condition of housing and ease of navigation between home and wider neighbourhood/community space. Place and home have been shown to be important non-determinants of health ( Field & Briggs, 2001; Macintyre *et al*., 2003) and yet have limited expression in Andersen’s model. The model is primed to further develop these domains to reflect the life course more fully.

In the review of Andersen’s model by Guilcher *et al*. (2012), it is recommended that ‘home layout’ be included in future access studies for people with spinal cord injury. Applications of Andersen’s model have incorporated the idea of distance travelled to health services, but Andersen does not specify access to transport as a factor in influencing HSU.

Culture is another aspect of the life course incorporated into HSU frameworks. Babitsch *et al*. (2012) include culture as an additional domain when mapping previous applications of Andersen. Culture is not a specific domain in the Andersen model but can manifest under the social structure aspect of the pre-disposing factors. In addition, cultural factors have been identified as enabling in application of the Andersen model. It is important to consider that the role of other people and cultural and social norms, may in fact be prohibitive to using health services and engaging with the health system.

This scoping review has identified a number of frameworks that illustrate how elements of the life course can be incorporated into the study of health service utilisation, although no framework included all aspects. The additional frameworks identified in this review, demonstrate commonality in variables with the Andersen model. These commonalities are illustrated in Table 5 (column 2).

The unique variables identified in these frameworks have a strong propensity towards the human agency aspect of the life course and position the individual as a more active participant in their health care and life situation. There is also added focus on the timing of care and temporality overall which is not easily identified in Andersen and which the current analysis considers absent.

### The life course and HSU

The WHO (2002) perspective of the individual as an active participant in their health and healthcare, is evidenced in the findings of this scoping review. The identified frameworks positioning the individual at the centre of their own lives and in particular their health experience. Choice, decision making and the individual demonstrating agency over their health experience and HSU experience is explicit.

Selecting among competing priorities is a judgement that individuals face in everyday life and in the healthcare context. Such competing decisions also exist at a health system and health policy level. This cost/benefit judgement will implicitly impact on if, when, how and the type of health services accessed by individuals and decisions to forgo required health services. Influencing factors at these decision points include the social network and connections that the individual has, as well as family, community and broader cultural and societal beliefs and values.

In particular, the role that environmental factors play in the HSU experience of minority or more vulnerable populations, such as health equity, healthcare reaching and cultural and social capital ( Nauenberg *et al*., 2011) is highlighted, and includes issues of discrimination and stigma as well as legal status of minority groups within the community ( Lee *et al*., 2009).

Social interconnectedness ( Levy *et al*., 2014), psychosocial ( Nauenberg *et al*., 2011) and affective supports ( Witucki & Wallace, 1998) are also important influences on HSU. Availability of supports, in addition to the availability of services, is another strong theme within the reviewed frameworks and align with the interdependency and relationship domain of the life course. Andersen’s predisposing domain could incorporate social relations, but the context or nature of the role of social relations in HSU is unclear within the model.

The importance of the provider-patient relationship and ability to communicate and establish rapport in the HSU process was noted and highlighted, particularly for immigrants who were non-English speaking ( Jo *et al*., 2008). Broader issues of human rights, feminism, and stigma are included within the interdependency domain as they impact on the status and roles an individual has within a given society and how others in society interact with the individual.

Cultural beliefs and folk values also present a type of relationship and interdependency that informs HSU for some people. The influence of the individual’s support networks and the inter-relationship between the individual and the broader cultural belief system within which they live or have lived has been shown to influence type of health care, such as decision to use traditional or complementary health services compared with mainstream health services. The heightened focus on cultural factors and distinguishing culture from community is an important addition.

Andersen does not address cultural factors as distinct from community, however reviews of the Andersen model have identified the application of culture in the use of the model ( Babitsch *et al*., 2012) under the rubric of contextual factors. Similarly, social relationships are not explicitly mentioned in the Anderson model, but are implied through its application.

Adopting a life course perspective to HSU enables a disaggregation of the influence of the family unit, which changes over the trajectory of the life course. This impacts on the use of health services through availability of support from other people to access services. Others knowledge, attitudes and experience of the health system, predisposition to mainstream or alternative, complementary or culturally specific values, and beliefs of appropriate health system to engage with, can also affect HSU. The inclusion of living status by Toloo *et al.* (2013) in examining HSU, that is whether the person lives alone or with someone else (partner, children, family etc.), highlights the link between place and people.

Under location, the need to include a more refined and ‘local’ place variable to better understand HSU is highlighted. Though the location of the health care interaction needs consideration, so does the type of housing, structural issues, living situation, neighbourhood and community factors. These place variables, which were identified and differ to that of site of health service and distance to travel, which are included in Andersen, are shown to be useful additions. Transportation ( Messer *et al*., 2013; Shaw *et al*., 2013) highlights the relevance of how an individual can navigate the space they live in to access services. Alborz *et al*. (2005) also indicate service setting as an important contributor to HSU, specifying the need to set characteristics of opening times and waiting times in understanding use. In addition, the home setting and social connections within the home are also relevant locational factors. Where an individual lives and who an individual lives with, as well as how geographically close to networks of support they are, acknowledges the role of the social determinants of health ( Dahlgreen & Whitehead, 1991; Emerson *et al*., 2011).

Temporality is the fourth domain of Elder’s life course perspective but there is limited focus on time and the impact of life events over time in the frameworks. The absence of time illustrates the continued tendency to view health access at one point in time. Of the four models with some reference to temporality, only one explicitly named time. However, life changes and life events were identified in others as potential trigger points for health service use. The inclusion of a time variable acknowledges that health is impacted by early life events as is current access and use of appropriate services ( Norris & Aiken, 2006). HSU is not static. It is a complex process, involving multiple service interactions at any one time. Interaction with one element of the health system may be a trigger for later interactions with the same or other aspects of the system. Hser *et al*. (2007) introduces the concept of duration of use in addition to frequency and highlights the importance of capturing HSU data over multiple time points.

As noted above, not all of the frameworks identified are traditional HSU models but have been applied to understand, explain or predict service utilisation in the health sector. Of note, the health belief model was widely used when the service focus was screening and health checks, with studies of emergency care and ambulance service use more likely not to use a framework. It could be implied that the move beyond HSU frameworks is an indication of the limits of current HSU frameworks, including Andersen.

The identified models of HSU add to the understanding of HSU by including the individual as an active participant in health and utilization of services. This is demonstrated through the focus on decision-making and choice elements. The individual utilization of healthcare does not exist in isolation. HSU is demonstrated as informed by the geographical, social, temporal and cultural context within the person’s life, and is more deeply explored in these models.

In addition to the differences between these models and the Andersen model, some similarities were identified, with many of the reviewed frameworks including variables that map to the traditional domains of the Andersen model; predisposing, enabling, need, health system characteristics and outcome variables. Most notable was the prevalence of predisposing and enabling variables. However nearly 40% (n=27) of the frameworks included variables that did not easily map to Andersen’s framework. See Table 5, column 3 to identify the variables that were unique to other frameworks.

## Conclusion

The dominant HSU model, developed by Andersen, does not offer a life course approach to the examination of HSU. The current review highlights that researchers are exploring aspects of the life course to understand how and why people choose to use health services. The role of personal choice and autonomy; the application of choice, and to a lesser extent control, in HSU frameworks have been highlighted.

This application of a life course framework to HSU not only helps to categorise and understand the additional variables/domains included within the reviewed frameworks, but also sheds light on some limitations of the Andersen framework. In particular, extending the understanding of HSU beyond the health system, the individual and community at one point in time, to encompass HSU over changing time and space is a particularly important learning.

Health and HSU are framed by where one lives and who one lives with and vice versa; choice of location or home is influenced by one’s need for service. This systematic review of frameworks highlights how the individual is positioned at the centre of their own lives and in particular their health experience. The position of the individual in this and the extent of choice and autonomy is impacted by and impacts on both health and home.

None of the identified models took an explicit life course approach or incorporated all four elements of the life course. Thus, the reviewed frameworks provide evidence for how elements of the life course can be included in frameworks to understand HSU but further work is needed to develop a model that includes all life course domains explicitly and comprehensively. A limitation of HSU models overall is the failure to recognise the impact of earlier life events on health and HSU. Previous health system interactions and knowledge of HSU impact on current and future HSU as well as other people’s previous HSU experience.

Important considerations in the utilisation of services by specific sub-groups of the populations should inform the content and structure of any future framework applied. The comprehensive examination of domains of the HSU frameworks has highlighted a range of factors that can potentially enhance the ability to plan the delivery and financing of future health care. Overall the issues and experience of the individual across the life course will likely impact on the ease with which required services are accessed at the appropriate time and place. Greater application of these domains within general HSU frameworks could inform future HSU models for people currently more susceptible to health inequalities across the life course.

## Limitations of the review

Due to the inclusion criteria for this review, it is likely that not all frameworks that have been used for the study of HSU were identified. Frameworks used for the specific HSU experience of children, low-middle income countries and people with mental health difficulties are not covered in this review. Some of the identified frameworks may be applied in these contexts but there may also be additional frameworks which have not been identified in this review.

The quality of the studies using frameworks was not assessed. The aim of this review was to identify the range of frameworks, other than Andersen’s Behavioural Model, that have been applied to the understanding of HSU. The study design and methodological issues were not of relevance to this question as the focus was the presence and use of a framework.

## Data availability

Zenodo: A Narrative synthesis Analysis of Life Course Domains with Health Service Utilisation Frameworks. https://doi.org/10.5281/zenodo.2613425 ( O’Donovan *et al*., 2019).

This project contains the following data:

*Underlying data*

- HSU_Narsyn_70FWs: The complete list of the 70 frameworks

*Reporting guidelines*

- PRISMA-ScR checklist
- PRSIMA flow diagram

Data are available under the terms of the Creative Commons Attribution 4.0 International license (CC-BY 4.0).
