# Supplementary material for: A narrative synthesis scoping review of life course domains within health service utilisation frameworks
Source: HRB Open Res. 2019 Mar 29;2:6. [Version 1] doi: 10.12688/hrbopenres.12900.1 (PMC7140772; doi:10.12688/hrbopenres.12900.1)
Supplement: Peer review history

# Peer review: Reviewer response for version 1

**DOI:** 10.21956/hrbopenres.13975.r26640

**Published:** 2019-07-29

**Authors:** Mattijs Numans

**Affiliations:** 1 Department of Public Health and Primary Care, Leiden University Medical Centre, Leiden, The Netherlands

This is a very thoroughly carried out systematic review of existing literature on healthcare utilisation from a life course perspective. I have no relevant concerns on the methods and content nor on conclusions, it was very interesting to read. Although shortening would be attractive from a readers perspective, I don't believe much of the content can be missed to understand the conclusions. However, maybe further editing (by native speakers, which I am not) might help. I would be willing to accept this manuscript for indexing as is.

I confirm that I have read this submission and believe that I have an appropriate level of expertise to confirm that it is of an acceptable scientific standard.

# Peer review: Reviewer response for version 1

**DOI:** 10.21956/hrbopenres.13975.r26563

**Published:** 2019-04-24

**Authors:** Duncan Chambers

**Affiliations:** 1 School of Health and Related Research (ScHARR), University of Sheffield, Sheffield, UK

The paper is a scoping/mapping review rather than a full systematic review. As such, some elements of systematic review methodology, for example assessment of risk of bias in included studies, are omitted. Relevant reporting guidelines were followed and the results are available in supplementary files. The authors don’t say explicitly whether a protocol was developed in advance and I suggest that this could be clarified. The literature search was thorough, although the search strategy was basic, leading to a large number of records to be screened.  

 The inclusion criteria were clear, although it was a bit of a stretch to fit them to a PICO-type structure (Table 1). Many other review question formulations are available, for example the authors might like to look at BeHEMoTh ( **Be**haviour; **H**ealth context; **E**xclusions; **M**odels **o**r **T**heories; Booth and Carroll; 2015 ^1^).

 Methods for identifying frameworks and mapping them against the Andersen model were clearly reported. A minor point is that Figure 2 moves from counting articles to counting frameworks at level 4. How many articles do the 164 frameworks represent?

 It may be unavoidable but the process of meta-synthesis comes across as a bit of a ‘black box’ (‘a process of sense-making and developing a cohesive structure’). Can the authors enlarge on this at all?

 The presentation and discussion of the review findings follow logically from the structure of the life course domains and Andersen’s model. The main conclusion about the need to develop a new model also follows logically from the review findings. The conclusion in the abstract that applying such a framework would be of benefit to researchers etc. may be overstated as this review is descriptive rather than evaluative.

I confirm that I have read this submission and believe that I have an appropriate level of expertise to confirm that it is of an acceptable scientific standard.

## References

[1] : Systematic searching for theory to inform systematic reviews: is it feasible? Is it desirable?. *Health Info Libr J*.2015;32(3) : 10.1111/hir.12108 220-35 10.1111/hir.12108 26095232
